# From Dermal Patch to Implants—Applications of Biocomposites in Living Tissues

**DOI:** 10.3390/molecules25030507

**Published:** 2020-01-24

**Authors:** Karolina Papera Valente, Alexandre Brolo, Afzal Suleman

**Affiliations:** 1 Department of Mechanical Engineering, University of Victoria, Victoria, BC V8P 5C2, Canada; kvalente@uvic.ca; 2 Department of Chemistry, University of Victoria, Victoria, BC V8P 5C2, Canada; agbrolo@uvic.ca

**Keywords:** biocomposites, scaffolds, biocompatibility, bone regeneration, orthopedic implants, wound healing, tissue engineering

## Abstract

Composites are composed of two or more materials, displaying enhanced performance and superior mechanical properties when compared to their individual components. The use of biocompatible materials has created a new category of biocomposites. Biocomposites can be applied to living tissues due to low toxicity, biodegradability and high biocompatibility. This review summarizes recent applications of biocomposite materials in the field of biomedical engineering, focusing on four areas—bone regeneration, orthopedic/dental implants, wound healing and tissue engineering.

## 1. Introduction

Composite structures consist of two or more material components. Composites can be engineered or natural, as is the case of bone structures. Bone is a natural composite composed of hydroxyapatite, a ceramic substance, combined with fibers of collagen, a soft (low elastic modulus) polymeric material. While hydroxyapatite provides the strength needed to support the body, ceramics are well known for their brittle character. Combining hydroxyapatite with collagen creates mineralized collagen fibril, that displays not only high strength due to the hardness of hydroxyapatite but also flexibility due to the presence of collagen. Therefore, composites show enhanced properties when compared to their individual material components. Given the heterogeneous characteristic of the composites, it is important to have good mixing/adherence between the components since lack of adhesion may result in structural failure [1].

Biocomposites display high biocompatibility and, consequently, can be used in contact with living organs and tissues [2]. In addition to compatibility, when applicable, degradable biocomposites are required to display degradation rates that closely match the renewal rate of tissues being replaced; non-toxicity; and enhanced cell interaction/growth.

**Figure 1:**
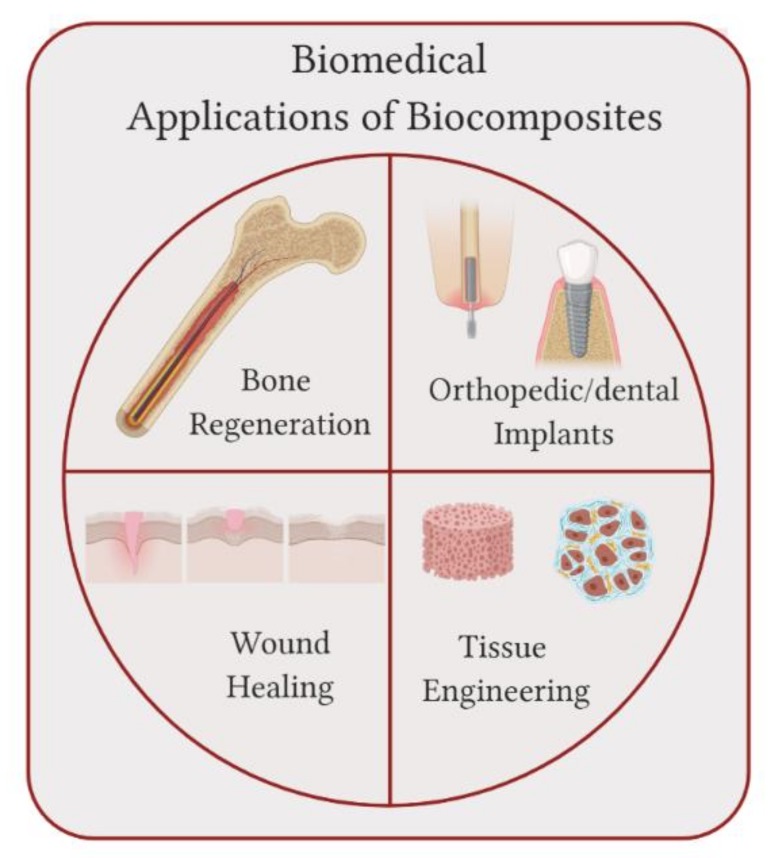
Biomedical applications of biocomposites: biocomposites can be applied to bone regeneration, orthopedic implants, wound healing patches and to tissue engineering.

This review summarizes some of the recent work on biocomposites with application to the fields of bone regeneration, orthopedic/dental implants, wound healing and tissue engineering, as illustrated in Figure 1. In the case of bone regeneration, biocomposites composed of hydroxyapatite in combination with different types of polymers have been investigated. Synthetic bone scaffolds displayed not just high strength but also porous structures, allowing the incorporation of bone-related cells, such as mesenchymal stem cells, in the matrices, as described in Section 2. In the case of orthopedic/dental implants, since metals display higher elastic moduli values than bone, biocomposites composed of polymer and ceramics have been explored as substitutes to metal grafts, as examined in Section 3. Section 4 discusses biocomposites that can also be applied to wound healing. Since degradation needs to match skin regeneration rate while maintaining a moist environment, the use of biocomposites enhances the possibility of finding a combination of materials that are able to accomplish the design requirements for a wound dressing. Finally, Section 5 presents the application of biocomposites that extend to tissue engineering where a combination of biomaterials is used to repair and restore damaged tissues using artificial scaffolds.

## 2. Bone Regeneration

Bone defects may arise as a consequence of trauma, cancer, congenital bone defects, infections and bone-related diseases such as osteoporosis. Bone grafts can be used to address bone loss, with autografts and allografts. While autografts use the tissue of the patient, allografts use tissue from another patient. However, both approaches present disadvantages such as limited supply, risks of disease transmission and infections [3]. Therefore, there is a need for development of synthetic bone scaffolds. Replacement of damaged bone with scaffold is one of the main goals of tissue engineering, in order to regenerate functionality on the impaired area. Ideally, scaffolds should have a highly porous three-dimensional structure in order to allow not just cell growth and communication but also flow of nutrients [4]. In addition, in order to replace a tissue or damaged area, scaffolds need to be biocompatible and have a controllable degradation rate that closely matches the cell growth of the tissue, allowing cell attachment, proliferation and finally, replacement of the scaffold by tissue growth. Lastly, mechanical properties of the scaffold, such as stiffness, should be similar to the characteristics of the tissue being repaired [5].

**Figure 2:**
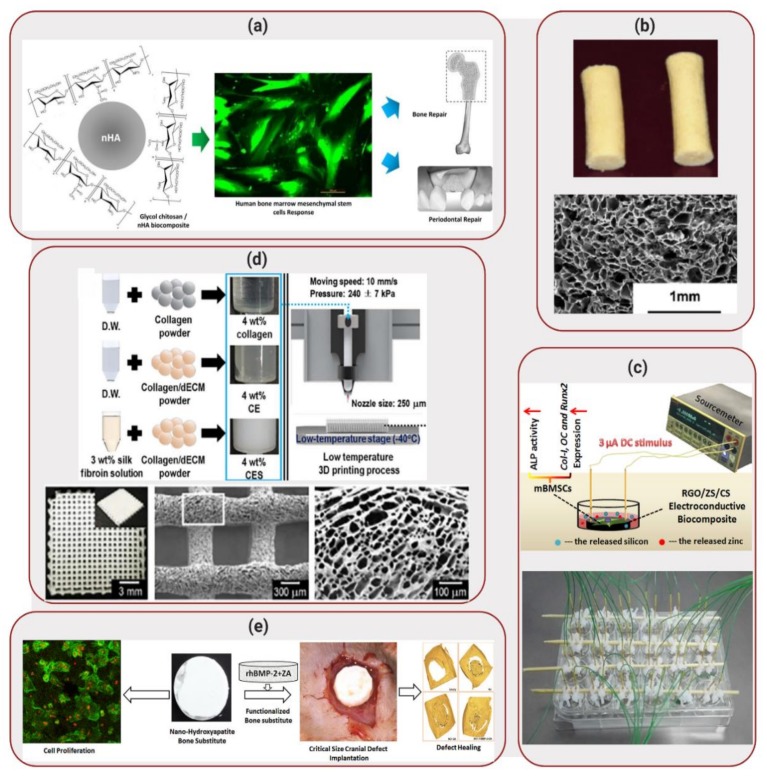
Applications of Biocomposites to Bone Regeneration: (**a**) Synthesis of glycol chitosan biocomposite matrices with nanohydroxyapatite. HBMS cells seeded on the matrix showed enhanced cell viability (live cells labelled in green). Reproduced with permission from Reference [10]. (**b**) Biocomposite cryogel pellets (top) composed of silk fibroin, chitosan, agarose and hydroxyapatite. The cryogels displayed a microporous structure, as illustrated by scanning electron microscope (SEM) image (bottom). Reprinted with permission from Reference [11]. (**c**) Electrical stimulation of reduced graphene oxide (RGO)/ zinc silicate (ZS)/ calcium silicate (CS) electroconductive biocomposite scaffolds. Reprinted with permission from Reference [15]. (**d**) Fabrication process of 3D-orinted scaffolds using a low temperature process. The scaffolds, composed of collagen, silk fibroin and decellularized extracellular matrix (dECM), displayed high porosity. Reprinted with permission from Reference [26]. (**e**) Development of nanohydroxyapatite bone substitute scaffolds, with release of bone morphogenic protein (BMP). Scaffolds were implanted in rats with cranial defects. Reprinted with permission from Reference [32].

Bone is basically composed of hydroxyapatite, a stable form of calcium phosphate and collagen, the most abundant protein found in the human body [6,7,8]. Consequently, biocomposite scaffolds applied to bone tissue regeneration are more frequently composed of a combination of a ceramic component, due to their high mechanical strength and a polymer component, to allow flexibility [9]. Dumont et al. [10] selected nanohydroxyapatite and glycol chitosan as the ceramic and polymeric components, respectively, of the developed biocomposite. Considering that hydroxyapatite is a major component of bone together with collagen, the addition of this ceramic to the biocomposite did not just add mechanical strength but mimicked the bone environment more closely. In this study, nanohydroxyapatite particles were synthesized using glycol chitosan as the ligand. Membranes of the composite material were created by pouring the polymer/calcium phosphate mixture into plastic molds and allowing them to dry. During scanning electron microscopy (SEM) analysis of the membranes, a homogeneous distribution of the nanohydroxyapatite particles was observed, resulting in uniform dispersion with the polymeric matrix. In order to verify biocompatibility of the composite membranes, live/dead and MTT assays were performed using human osteosarcoma (Saos-2) and human bone marrow mesenchymal stem cells (HBMS) cell lines, displaying high cell viability (~100%) and, consequently, non-toxic behavior. In addition, HBMS cells displayed good cell adhesion, spreading and proliferation on the surface of the biocomposite, as shown in Figure 2a. The positive results obtained in this study demonstrated that the developed biocomposite scaffold offers valuable applications such as moldable membranes for orthopedic surgeries. While further in vivo investigation is required to realistically assess the application of this biocomposite, so far this material has shown to be a good potential candidate in the field of tissue regeneration.

In a similar approach, Raina et al. [11] combined hydroxyapatite with different polymers (fibroin, chitosan and agarose) in order to develop a biocomposite cryogel scaffold. An additional ceramic material, bioactive glass, was also added to the composite, considering that bioactive glass is known to form hydroxyapatite-like crystals when in contact with body fluids, having the potential to enhance biocompatibility. Scaffolds displayed a macroporous architecture, as illustrated in Figure 2b and high elastic modulus (350–700 kPa). Mouse myoblast cells (C2C12) and rat mesenchymal stem cells (MSCs) were seeded on the scaffold and demonstrated good cell proliferation until day 45 of seeding (increase in MTT absorbance from ~0.5 and ~1.0 on day 0 to ~2.0 and ~2.5 on day 45, respectively). In addition, the cells also demonstrated elevated alkaline phosphatase (ALP) activity levels, cueing towards osteogenic differentiation. In order to develop a functionally active scaffold, bone active agents such as recombinant human bone morphogenic protein (rhBMP) and zoledronic acid (ZA) were also added to the cryogels. While BMPs are known to not just enhance osteoinduction but also cause bone resorption, the addition of ZA was performed in order to prevent bone resorption as demonstrated previously [12,13,14]. Proliferation of mouse osteoblastic cells (MC3T3) on the scaffolds containing rhBMP and ZA was performed to evaluate the effect of the bone active drugs on the maturation of the cells, resulting in a significantly higher production of ALP by the cells when compared to the scaffolds without active drugs. This scaffold was then implanted in the abdomen area of rats, between muscles. After 4 weeks of in vivo implantation, the scaffolds demonstrated bone formation (~0.1 mm^2^ of area of bone in the absence of ZA compared with ~1.5 mm^2^ in presence of ZA in the scaffolds), indicating new bone growth onto the scaffold and, consequently, demonstrating potential as a replacement to bone grafts.

Considering that bone defects may also arise from diseases such as osteoporosis (predominant disease due to the aging population), Xiong et al. [15] explored the development of an electroconductive biocomposite for stimulating bone regeneration under osteoporosis conditions. Previous promising results have demonstrated that silicate-based biomaterials are able to be used in the enhancement of osteoporotic bone regeneration [16,17,18,19,20]. Consequently, the biocomposite developed by Xiong et al. [15] was composed of different silicates organized in three layers—a calcium silicate (CS) substrate base, a zinc silicate (ZS) middle layer and a reduced graphene oxide (RGO) top layer. Zinc and silicon are key trace elements in the bone, known to enhance bone regeneration [21,22]. In addition, calcium silicate is known to provide good osteoinductivity [23,24].

In vitro osteogenesis of the electroconductive biocomposite was evaluated in presence of mouse bone marrow stem cells (mBMSCs) seeded at the surface of the scaffold, while 3 μA was applied to stimulate the cells (1 h per day of stimulation time), as illustrated in Figure 2c. The results displayed high ALP activity, demonstrating a total of protein content of ~16 U/mg in the biocomposite compared with ~8 U/mg in the control (cells seeded on a cell culture plate). In addition, considering that vascularization during bone formation is important to deliver nutrients to the affected area, the angiogenic capability of RGO/ZS/CS extracts was also evaluated. Human umbilical vein endothelial cells (HUVEC) were cultured in an ECMatrix gel with and without the RGO/ZS/CS extract. In the absence of the extract, the HUVECs formed branched nodes, indicating angiogenesis. However, in the presence of the biocomposite extracts with different dilution ratios, mesh-like circle structures were observed, indicating that HUVEC proliferation increased and expression of angiogenesis-related genes was promoted. These results indicated that the electroconductive biocomposite developed by Xiong et al. [15] demonstrated superior in vitro osteogenesis and angiogenesis performances, displaying a large potential for stimulating osteoporotic bone regeneration. Although promising, application of this biocomposite to osteoporotic fracture healing will require further in vitro and in vivo studies, moving beyond the seeding of cells at the surface of the scaffolds.

Considering the use of micrografts, Rodriguez and Baena et al. [25] investigated the combination of poly(lactic-co-glycolic acid) (PLGA) and hydroxyapatite to periosteum-derived micrografts in the enhancement of bone formation in sinus lift procedure (surgical procedure to augment amount of bone in the upper jaw). Micrografts supplemented with PLGA and hydroxyapatite were implanted in 24 patients. After 4 months, ossification process was higher in the biocomposite-micrografts when compared with control groups (biocomposites in absence of grafts). In addition, a significant amount of mineralized tissue was observed, displaying the efficacy of the biocomplex composed by micrograft and biocomposite in bone formation.

While many biocomposite scaffolds are routinely fabricated based on molding techniques, Lee et al. [26] explored the 3D printing fabrication of a porous scaffold composed of collagen, silk fibroin and decellularized extracellular matrix (dECM) to be used for bone tissue regeneration. The addition of dECM aimed to increase cellular activities, due to its high biocompatibility. The scaffolds were 3D-printed at low-temperature (−40 °C), followed by freeze-drying prior to crosslinking of collagen components at room temperature. The scaffolds displayed high porosity, as illustrated in Figure 2d, with pore sizes larger than 300 µm that is required in order to enhance bone formation [27]. MC3T3 were seeded on the scaffolds and, after 7 days of culture, the cells displayed high ALP expression (1.34-fold compared to pure collagen scaffold) and greater calcium deposition (1.58-fold). In addition, high cell proliferation was observed, with an increase in cell nuclei density from ~260 cells/mm^2^ for pure collagen to ~380 cells/mm^2^ for the scaffolds. These results illustrated the potential of this 3D-printed biocomposite as a biomedical scaffold for hard tissue regeneration. Further analysis of application of the biocomposite scaffolds will possibly require in vivo implantation and verification of bone tissue regeneration. In addition, large scale production of 3D printed scaffolds will be necessary in order to feasibly apply this biocomposite to the field of bone regeneration.

Also focusing on an alternative fabrication method for biocomposite scaffolds, Gautam et al. [28] developed a 3D hexagon boron nitride-based biocomposite by spark plasma sintering. This technique uses pressure-driven powder consolidation based on a pulsed electric current passing through the sample [29], allowing densification of ceramic powders [30]. The biocomposite developed was composed of a 3D hexagon boron nitride nanostructure interconnected with boron trioxide. The structures demonstrated high stability and high mechanical strength (281 MPa of compressive strength and 63 MPA of Young’s modulus). Osteogenic activity of the biocomposite was investigated by culturing mice calvarial osteoblast cells for 21 days on the structures. Increased mineralization was verified on the biocomposite (up to ~91% increase in mineralization when compared with control samples). High cell culture performance in addition to good mechanical properties indicates that this biocomposite shows promising applications to the field of bone implants.

Considering that bone defects may also arise as a consequence of trauma, when an injury happens in the skull, a cranioplasty may need to be performed. This surgery consists of repairing the bone defect using materials such as metals, ceramics, polymers and autologous bone grafts [31]. Focusing on finding alternatives to current bone grafts used in cranioplasty, Teotia et al. [32] developed a hydroxyapatite bone substitute for enhanced cranial bone regeneration. The biocomposite cement was composed of nanohydroxyapatite, in order to mimic the nanotopography of bone, calcium sulfate, a based material used in bone fillers [33] and bioactive molecules (BMP and ZA), to provide osteoinductive properties and enhance bone formation. In addition to allowing growth of MC3T3 cells when cultured on the discs-shaped cement, these cells displayed high levels of ALP, illustrating that the biocomposite was osteoinductive and supported cell differentiation.

Circular discs were also prepared out of the biocomposite cement for surgical implantation in rats with cranial defects, as illustrated in Figure 2e. Micro-CT analysis were carried out at 8 weeks and 12 weeks after the implantation of the biocomposite. The results indicated that the amount of mineralized volume was significantly higher in the animals implanted with the biocomposite cement (13.0 mm^3^) than in the control groups (6.5–9.0 mm^3^).

Scanning electron microscope (SEM) images were performed on the post sacrifice rat bone samples from the defect site, illustrating a high amount of mineralized tissue in addition to bone formation. Therefore, the biocomposite cement displayed biocompatible properties in addition to supporting bone formation, with osteoinductive properties due to the addition of bone anabolic and anticatabolic agents (BMP and ZA), showing high potential to be used in cranioplasty and similar bone defects. While promising in vivo results were presented, further in vivo investigation with longer time frames are still necessary.

## 3. Orthopedic Implants

Biocomposites can also be applied to the field of orthopedic implants. Orthopedic materials are normally based on metal alloys such as titanium and stainless steel, because of their biocompatibility and their mechanical properties. However, there is a large concern around the release of metal particles in the body, as has been observed with cobalt-chromium based alloys [34,35]. In addition, some metallic implants show a mismatch in terms of elastic modulus, displaying higher elastic modulus than bone, which can result in an increase of stress in the implanted area and also in bone resorption [36]. Therefore, alternative synthetic polymers have been explored, such as non-degradable polymer polyetheretherketone (PEEK), that displays elastic modulus in the range of 3–4 GPa, which is much closer to the elastic modulus of human cortical bone (18 GPa), especially when compared with the elastic modulus of metallic implants (in the range of 110 GPa) [37].

**Figure 3:**
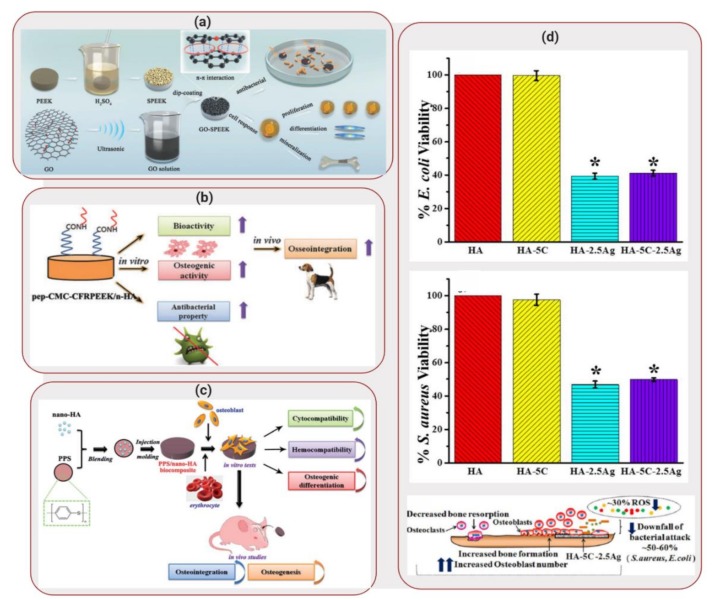
Applications of Biocomposites to Orthopedic/Dental Implants: (**a**) Schematic illustration of fabrication process of graphene oxide- polyetheretherketone (PEEK) scaffolds. Reproduced with permission from Reference [39]. (**b**) Schematic illustration of preparation of peptide-decorated carbon reinforced PEEK (CFRPEEK) /nanohydroxyapatite biocomposite and its in vivo osseointegration. Reprinted with permission from Reference [41]. (**c**) Schematic illustration of preparation of PPS/nanohydroxyapatite biocomposite and its in vitro/in vivo performance evaluation. Reprinted with permission from Reference [42]. (**d**) Viability responses of *E. coli* (top) and *S. aureus* bacteria when seeded on hydroxyapatite scaffold reinforced with ceria and silver nanoparticles. Representation of aftereffects of this scaffold when hFOBs were seeded on it. Reprinted with permission from Reference [44].

Even though PEEK presents mechanical properties closer to those found in bone, applications of this synthetic polymer is still limited due to its bioinertness [38]. To overcome these limitations, Ouyang et al. [39] developed a biocomposite composed of PEEK and graphene oxide. Graphene oxide contains an abundance of hydroxyl, carboxylic and epoxide functional groups, creating reactive sites for functionalization which has the potential to enhance osteogenic differentiation of stem cells [40]. Therefore, Ouyang et al [39] developed graphene oxide self-assemblies on micro/nanoporous PEEK, illustrated in Figure 3a. The introduction of porosity into the PEEK structure has been shown to enhance the interaction with bone, promoting ingrowth of tissues into the material. PEEK samples were sulfonated by immersion into a sulfuric acid bath and sonicated in order to obtain a uniform porous structure. The samples were then soaked in a solution containing graphene oxide and water, followed by a drying process. SEM images demonstrated that the biocomposite contained pores between 200 and 300 nm (nanostructure surface). In addition, contact angle measurements showed a decrease from 91.2° for bare PEEEK to 47.7° for graphene oxide-PEEK, displaying an increase in hydrophilicity with increase of graphene oxide concentration.

Antibacterial tests were carried out by incubating the biocomposites with a solution containing *Escherichia coli* (*E. coli*) and *Staphylococcus aureus* (*S. aureus*). Live/dead fluorescence images were obtained to evaluate the viable state of the bacteria. A reduction of 77.3% of *E. coli* in graphene oxide-PEEK was observed, when compared to sulfonated PEEK. However, *S. aureus* displayed an upward tendency that could have been caused by the hydrophilicity property of the biocomposites. Adhesion, proliferation, morphology change and ALP activity level were investigated by seeding human osteosarcoma MG-63 cells on the graphene oxide-PEEK samples. Cells displayed a better spread over the substrate area, indicating that graphene oxide can provide a better environment for cell attachment and proliferation when compared to bare PEEK. In addition, cells exhibited high ALP activity in the biocomposites, followed by an expression of osteogenesis related genes. These results indicated that the surface modification of PEEK with graphene oxide was promising, showing that this biocomposite is a good bone replacement candidate for implantable materials.

In a similar approach, Xu et al. [41] investigated the impact of modifying carbon reinforced PEEK (CFRPEEK) with nanohydroxyapatite, carboxymethyl, chitosan and bone forming peptide. CFRPEEK is an interesting alternative to metallic implants due to its mechanical properties and non-toxicity. However, similar to PEEK, CFRPEEK applications are hindered due to its bioinertness. Therefore, the addition of different components by surface modification was performed in order to produce a biocomposite with enhanced anti-bacterial behavior and osseointegration. Powder nanohydroxyapatite was mixed with carbon fiber and PEEK and injection molding was used to fabricate the biocomposite scaffolds, as illustrated in Figure 3b. Sequentially, substrates were immersed in a solution containing carboxymethyl chitosan and finally, reacted with bone forming peptide solution overnight. Contact angle measurements displayed a decrease in water contact angle from 76 ± 1° (CFRPEEK-nanohydroxyapatite) to 18° for the biocomposite, illustrating that bone forming peptide was successfully grafted on the surface of the substrates due to its super-hydrophilic nature.

The antimicrobial activity of the biocomposites were assessed by incubating the substrates with *S. aureus*. A reduction of ~30% on the amount of viable bacteria was seen on the functionalized substrates compared to pristine CFRPEEK-nanohydroxyapatite. In addition, cell adhesion and proliferation were investigated by seeding human mesenchymal stem cells (hMSCs) to the substrates, considering that cell adhesion plays a major role in tissue integration, while cell proliferation can be correlated with bone formation. The highest cell adhesion and proliferation was observed in the modified biocomposites, illustrating a favorable environment for cell. In addition, high ALP activity was seen on the biocomposite substrates (~350 U/g compared to ~220 U/g on pristine substrate), indicated osteogenesis.

In vivo animal studies were conducted in order to assess osseointegration. The substrates were implanted into the cancellous bones of the tibia marrow cavity of beagles, in which hMSCs can be found abundantly. After 4 weeks, the implanted substrates demonstrated a large quantity of newly formed bone. Therefore, the biocomposite not only provided a positive effect on osseointegration but boosted bone formation around the implant. In addition, the improvement of antibacterial activity combined with osseointegration showed promising potential for this bioactive material in orthopedic implants, considering that further in vivo investigation is assessed.

Deng et al. [42], on the other hand, explored the use of polyphenylene sulfide (PPS) as an alternative to the use of PEEK as potential biomaterial. PPS displays comparable mechanical properties to PEEK and shows a lower production cost. Consequently, it can become a more potent candidate as bone-grafting substrate than PEEK. Therefore, a biocomposite was developed composed of nanohydroxyapatite reinforced in PPD by injection molding, as shown in Figure 3c. Hemocompatibility tests were performed since the interplay between blood and the implant affect bone formation and healing. The biocomposites were incubated with rabbit blood and results demonstrated that the substrates showed low procoagulant properties, displaying good hemocompatibility. In addition, cell proliferation and osteogenic differentiation was investigated for human osteosarcoma MG-63 cells seeded in the biocomposite. Nanohydroxyapatite doped PPS displayed higher cell multiplication when compared to pure PPS (optical density value at 570 nm increased from ~0.5 to ~0.7), in addition to higher ALP activity expression (average optical density increases on day 14 from ~1.2 to ~2.0) and formation of aggregates showing bone-like structures.

In vivo assessment of bone regeneration was performed by creating defects in the middle of skull of rats and implanting nanohydroxyapatite doped PPS, bare PPS and a surgical titanium mesh. A higher concentration of blood vessel infiltration was observed on the biocomposite, illustrating the progress of angiogenesis. The implanted biocomposite also displayed compact de novo bone and better bone healing. In addition, regeneration of blood vessels demonstrated that the quality of newly formed bone in contact with the implants was higher for biocomposites when compared with bare PPS and titanium. These results illustrated that doped PPS displayed positive osseointegration and also enhanced bone maturation around the implanted area. Although these results are still preliminary, PPS-based biomaterials show promising applications in the orthopedic field.

Similarly, Asadullah et al. [43] explored the use of polyimide (PI) as an alternative non-degradable polymer to PEEK. PI was combined with tantalum pentaoxide (TO), an oxide that has shown promising orthopedic applications due to its biocompatibility. The biocomposite was fabricated by blending TO with PI through cold pressing and sintering, containing two different TO contents 25 *wt*% (TPC25) and 50 *wt*% (TPC50). Compressive strength measurements demonstrated enhanced compressive values for TPC25 and TPC50 (110 MPa and 130 MPa, respectively), when compared with PI alone (98 MPa). In addition to enhanced strength, in vitro apatite mineralization of the samples performed in simulated body fluid showed that apatite deposition was higher on TPC50 compared with TPC25, while no deposition was verified on PI surface. Rat bone mesenchymal stem cells (rBMSCs) were cultured on the surface of the samples. After 3 days, cells demonstrated high adhesion, proliferation and cluster formation on the surface of TPC50, compared with the other two substrates. In addition, ALP activity was also higher in TPC50. These results indicated that increasing TO content enhanced mechanical strength and created a rough surface, improving cellular response and, consequently demonstrating that the TPC50 biocomposite may be applicable for orthopedic implants.

Pandey et al. [44], on the other hand, explored not just the fabrication of a biocomposite scaffold but also the applicability of the biocomposite as a coating for metal substrates. The hydroxyapatite-based biocomposite was reinforced with ceria and silver nanoparticles. While ceria particles have shown to offer enzyme mimetic properties, silver displays antibacterial properties. Pellets of the biocomposite were produced by combining different concentrations of ceria and silver nanoparticles to hydroxyapatite. *E. coli* and *S. aureus* were seeded on the surface of the substrates and incubated for 4h. In absence of silver, similar adhesion was obtained for both bare hydroxyapatite and hydroxyapatite with ceria (viability close to 100%). However, in presence of silver, a decrease in viability was seen to ~40% and ~50% for *E. coli* and *S. aureus*, respectively. Silver is known to have antibacterial properties due to the ability of the silver ions to denature bacterial DNA [45].

Considering that serum albumin is the most abundant protein found in the blood [46], it is important to investigate albumin adsorption to the surface of the substrates. Therefore, substrates were incubated with bovine serum albumin for 24 h. An increase of 3.84 times was observed when compared adsorbed protein in the biocomposite with bare hydroxyapatite. The increase in adsorption can be related to protein adsorption on the silver surface but also to the increase in hydrophobicity [47]. In addition, human fetal osteoblasts (hFOBs) seeded on the substrates showed increased cell viability and activity in the biocomposites, indicating cytocompatibility of the synergistic effects of ceria and silver nanoparticles. These results illustrated that the ceria and silver nanoparticles doped hydroxyapatite can be used not just as a scaffold but also as an antibacterial bioactive coating for total hip replacement, since the biocomposite demonstrated not just enhanced mechanical properties but also enhanced osteoblast adhesion and proliferation, in addition to antibacterial properties. However, although not clear, further assessment of cell viability and antimicrobial activity in the same cell media needs to be performed in order to verify the potential of this biocomposite.

## 4. Wound Healing

Skin is not only the largest organ in the human body but it also acts as a defense barrier to the external environment. Damaged skin, such as open wounds, means that this defense mechanism is lost and the organism is now vulnerable to skin-related infections. Wound dressings can be used to accelerate the healing process, while avoiding dehydration and providing an antimicrobial environment to avert infection [48]. Materials used in the fabrication of wound dressings should be non-toxic, biocompatible and ideally accelerate cell proliferation [49]. When biodegradable, the degradation rate should be equivalent to the rate of wound repair [50]. Gas exchange is also an important parameter to be considered, since high carbon dioxide and low oxygen concentrations change the acidity on the wounded area, decreasing the healing process and making the wound more prone to bacteria growth [51].

**Figure 4:**
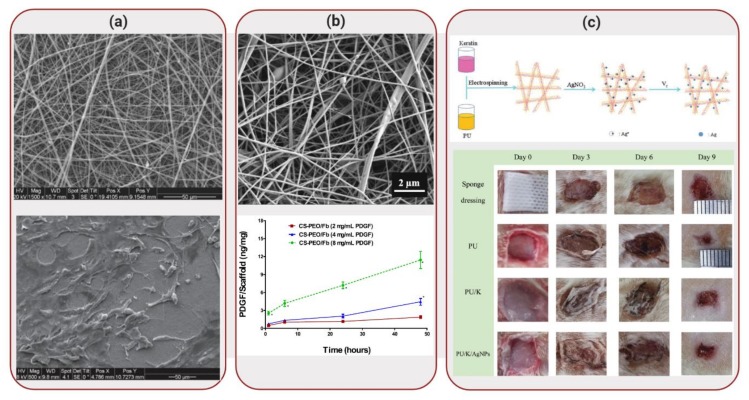
Applications of Biocomposites to Wound Healing: (**a**) SEM images of polycaprolactone-gelatin nanofiber scaffold (top) and morphology expression of human skin-derived precursor cells (hSKPs) cells on this matrix. Reproduced with permission from Reference [54]. (**b**) SEM image of fibrous scaffold composed of chitosan, polyethylene oxide, fibrinogen (top). Platelet-derived growth factor (PDGF) was released from the scaffolds, displaying a dose-dependent profile (bottom). Reprinted with permission from Reference [57]. (**c**) Schematic illustration of fabrication of polyurethane/keratin/silver nanoparticles scaffold (top). Wound healing tests were performed using different dressing materials, including a sponge dressing (control), in the back of rats (bottom). Reprinted with permission from Reference [63].

Hydrogels have been extensively used in wound dressing due to its biocompatibility and high water composition, maintaining a moist environment [52]. However, often a single component is not able to cover all necessary requirements to create an appropriate wound dressing. Synthetic polymers, for example, lack high cell affinity but display good mechanical properties. Natural polymers, on the other hand, are biocompatible and biodegradable but often present poor mechanical strength and limited shelf-life [53]. Considering these parameters, Anjum et al. [54] developed a nanofiber scaffold composed of polycaprolactone (PCL) and gelatin, as illustrated in Figure 4a. Even though PCL is a biodegradable polyester used in different medical applications [55], this polymer has shown reduced cell adhesion, due to a lack of cell-recognition sites. Gelatin, on the other hand, contains RGD (arginine-glycine-aspartate) motifs that enhance cell adhesion and proliferation. The combination of these polymers aimed to increase cell adhesion, proliferation and migration while exhibiting enhanced mechanical properties [56].

Electrospinning technique was used to obtain polycaprolactone-gelatin nanofibers. The growth of adult human skin-derived precursor cells (hSKPs) seeded on the nanofiber scaffolds was investigated in order to determine whether these nanofibers could be used in the delivery of cells into wounds. Nanofiber scaffolds displayed enhanced cell proliferation compared to bare polycaprolactone. By day 7, cells had almost completely covered the surface of the scaffolds, creating a monolayer, in which the cells started producing collagen. These results displayed that the combination of both polymers enhanced cellular activity, holding good promise in terms of delivering cells to injured skin areas.

In a similar approach, Yuan et al. [57] developed a fibrous scaffold composed of chitosan, polyethylene oxide and fibrinogen, in which platelet-derived growth factor (PDGF) was successfully incorporated, as illustrated in Figure 4b. PDGF helps to regulate matrix deposition and act as an initiator of the wound healing process [58]. Chitosan is a polymer derived from chitin that has been explored as a wound dressing material due to its antimicrobial properties [59]. However, since chitosan shows a polycationic nature, it is challenging to electrospin it. Therefore, chitosan was mixed with polyethylene oxide in order to improve electrospinability, while providing mechanical strength to the biocomposite. Fibrinogen works through its activated form (fibrin), which can be used as a temporary matrix for tissue repair and regeneration. However, fibrin lacks structural and mechanical integrity; challenges that were overcome by combining it with chitosan and polyethylene oxide. Addition of PDGF to the scaffolds demonstrated that, during in vitro degradation, the biocomposite exhibited a unique sustained release profile, promoting fibroblast migration. The chitosan-polyethylene oxide-fibrinogen scaffolds demonstrated a viable alternative to wound dressings, especially due to its capability to deliver bioactive PDGF. Although promising, feasible applications of this biocomposite will require in vivo investigation of PDGF delivery and enhancement of wound healing.

The antibacterial potential of silver can consecutively reduce the toxicity response to the scaffold and so Zhang et al. [60] incorporated silver nanoparticles on a collagen-alginate biocomposite. Alginate is a natural polymer extracted from brown seaweed and it has been extensively used in biomedical applications [61]. Although biocompatible, alginate shows low hemostatic performance [62]. Combining alginate with collagen, the most abundant protein in the human body and a major component of extracellular matrix, attempted to increase hemostatic function and promote cell proliferation.

Sodium alginate powder was dissolved in a silver nanoparticle solution and the mixture was added to a collagen solution, resulting in a collagen-alginate-silver nanoparticles blend, that was then casted into rectangular molds. Mouse embryonic fibroblasts were seeded in the scaffolds in order to evaluate biocompatibility of the biocomposite. When compared with the control group a relative viability of more than 72% was obtained for the cells when the concentration of silver was below 50 µM. Antibacterial activities of *E. coli* and *S. aureus* were also evaluated in the biocomposites. While composites without silver nanoparticles showed no antibacterial activity, the presence of silver nanoparticles inhibited the growth of both bacteria, exhibiting potent antibacterial activity and thereby displaying the strong potential of the biocomposite as wound dressings.

Also taking advantage of the antimicrobial properties of silver nanoparticles, Wang et al. [63] developed a biocomposite mat composed of polyurethane and keratin, in addition to silver nanoparticles. Keratin, a low-cost fibrous protein, was extracted from human hair in order to obtain S-(carboxymethyl)keratin. Considering that gas exchange is essential for wound healing, polyurethane is known for its ability to allow permeation of oxygen [64]. Combining these two polymers and fabricating the biocomposite by electrospinning allowed the development of polymeric fibers with high surface area and porosity, parameters required for wound dressing applications. Fibroblast NIH 3T3 cells seeded on the scaffolds displayed better cell growth in the biocomposites than in bare polyurethane. In addition, the biocomposites displayed high antibacterial activity against both *S. aureus* and *E. coli*. In vivo studies were performed by cutting 4 full-thickness circular wounds on the back of rats and applying the nanofibrous mats to the wounds and comparing the results with a sponge dressing, as illustrated in Figure 4c. In the sponge control group, inflammatory cell infiltration was obvious. On nanofibrous mats, on the other hand, the boundary of epidermis and dermis layers disappeared, while a reduction of inflammatory cells was seen. Therefore, in addition to cell growth stimulation, animal testing results showed that the biocomposite could promote wound healing. However, long-term in vivo studies are still necessary to fully test the potential of this biomaterial for skin repair.

More complex wound healing patches can be developed by incorporating sensing markers in order to monitor the healing process. Oxygen can be used as a healing marker, indicating tissue oxygenation and, consequently, providing valuable diagnostic information. Roussakis et al. [65] developed a theranostic biocomposite scaffold composed of collagen and dextran, containing an oxygen sensing component. Collagen-dextran mixture displayed high biocompatibility and biodegradability, while oxygen monitoring by in vivo phosphorescence imaging incorporated the ability to track the progression of the wound healing. The oxygen-sensing macromolecule (dendrimer) was incorporated into the collagen-dextran mixture, together with a green-emitting reference dye. In vivo evaluation of the performance of the biocomposite scaffold membrane was performed by incorporating the membrane with and without the oxygen-sensing marker in a wound made on the backs of diabetic mice. In vivo luminescence imaging was performed considering that as wound oxygenation increases, the rate of phosphorescence quenching increases, resulting in a decrease in phosphorescence intensity on the biocomposite. In vivo results demonstrated a large decrease in phosphorescence on days 0–1, followed by small variations until day 10. These results demonstrate the ability to observe and track oxygen levels using this composite membrane. Therefore, the incorporation of both therapeutic and diagnostic functionality into a single biocomposite, as in the case of the oxygen-sensing scaffold, cannot just enhance healing but also monitor the progression of the wound healing process.

## 5. Tissue Engineering

Biocomposites have also been applied to the field of tissue engineering. This field refers to the restoration of damaged tissues using artificial scaffolds [66]. Since the scaffold containing cells is supposed to enhance cell growth and regeneration of the damaged tissue, degradation of the scaffold needs to meet the regeneration rate of the damaged area. This is done by using biomaterials to mimic the extracellular matrix of tissues, that is mainly composed of collagen, elastin, fibrin and hyaluronic acid [67].

**Figure 5:**
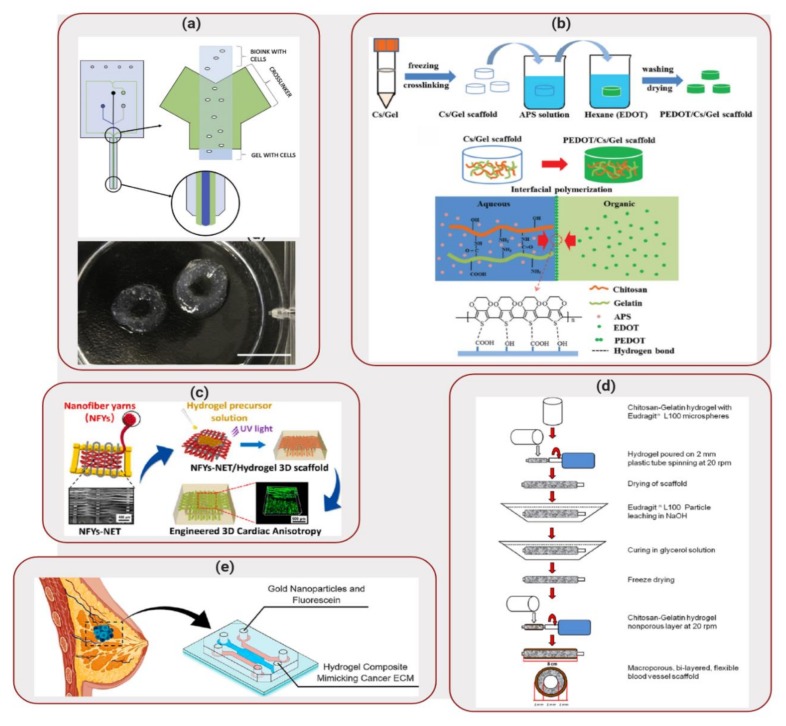
Applications of Biocomposites to Tissue Engineering: (**a**) Aspect biosystems’ DUO-1TM printhead (top) 3D printed glioblastoma multiforme (GBM) structure (bottom). Reproduced with permission from Reference [68]. (**b**) Schematic of the synthetic route to the PEDOT/Chitosan/Gelatin scaffold. Reprinted with permission from Reference [70]. (**c**) Design of nanofiber yarns network embedded in hydrogel to create 3D scaffold. Reprinted with permission from Reference [71]. (**d**) Preparation process of the macroporous bilayer tubular chitosan-gelatin scaffold. Reprinted with permission from Reference [84]. (**e**) Mimicking ECM in vitro using a biocomposite mixture inside of a microfluidic device. Reprinted with permission from Reference [89].

Lee et al. [68] developed a fibrin-based biocomposite that was used to create a 3D printed glioblastoma multiforme (GBM) model. The biocomposite ink was composed of fibrin, alginate and genipin. To crosslink the components of this bioink, calcium chloride, chitosan and thrombin were used. Aspect Biosystems RX1 printer was used to create the 3D printed model of GBM, in which U87MG human GBM cells were mixed with the bioink and the mixture was crosslinked at the exiting nozzle of the printhead, as illustrated in Figure 5a. High cell viability was observed after printing (~88%) and, after 12 days of culture of the scaffolds, ~86% of cell viability was obtained. In addition, cells also displayed the formation of spheroids within the scaffolds, indication the feasibility of using these constructs as drug screening models. The scaffolds were treated with a GBM-reprogramming cocktail (cocktail that has shown to reprogram GBM cells to neurons [69]). While elongated neurites were not seen, cells had impaired ability to proliferate throughout the scaffold and were not able to form cell-like spheroids. These results indicated that this 3D printed model could recreate a more accurate representation of GBM’s in vivo characteristics than 2D cell cultures, while using a fibrin-based biocomposite ink. Nevertheless, even though positive results were obtained, in vivo data still needs to be obtained, in addition to possible exploration of more complex 3D printed structures.

Also in the field of neural tissue engineering, Wang et al. [70] developed a conductive scaffold composed of chitosan, gelatin and poly(3,4-ethylene-dioxythiophene) (PEDOT) nanoparticles. Chitosan/gelatin porous scaffolds were immersed in a solution containing ammonium persulfate (APS) and EDOT monomer, as illustrated in Figure 5b. Final scaffolds displayed a porous structure, favorable for cell perfusion. In addition, the constructs displayed high electrical conductivity in hydrated state and in presence of cells, indicating that this biocomposite shows promising properties to be used as conductive material for tissue engineering. In combination with an appropriate biodegradation rate (~90% weight loss after weeks of incubation), cytotoxicity tests demonstrated that the degradation products did not alter cell viability. PC12 cells from rat adrenal medullary tumor were seeded on the scaffolds and demonstrated high adhesion, proliferation and spreading over the scaffold surface, displaying neurite growth. These results indicated that the PEDOT layer had a good biocompatibility and that the biocomposite shows potential to be used as an implant material for neural tissue engineering. Further *in vivo* investigation is still necessary to fully understand the potential of this biocomposite as an implant for neutral tissue engineering.

Applications of conductive scaffolds can also be extended to cardiac tissue regeneration. Wu et al. [71] developed a 3D hybrid scaffold based on aligned conductive nanofiber yarn networks. These networks were combined with a hydrogel shell in order to mimic the native cardiac tissue structure, as illustrated in Figure 5c, especially considering that heart muscles are dense quasi-lamellar tissues that display an anisotropic structure with multiple layers of extracellular matrix (ECM) and highly oriented cells [72,73]. The yarn networks were prepared using a weaving technique. First, a nanofibrous web was created by electrospinning a mixture composed of PCL, silk fibroin and carbon nanotubes. Surgical suturing threads were positioned with battlements pattern, forming a warp structure in which the fibers woven across, producing an interwoven network scaffold. Even though cardiomyocytes cultured on the surface of the network scaffold demonstrated high viability, aligned and elongated morphologies and a synchronous beating ability, the network structure was easily disrupted. Considering that the preparation of multilayer nanofiber yarns network is difficult, a core-shell approach was taken, in which gelatin methacryloyl was used to encapsulate the network. Cardiomyocytes seeded on this 3D structure exhibited long sarcomeres, indicating that this biocomposite structure enhanced cellular alignment, elongation and cardiomyocytes maturation. Therefore, this scaffold demonstrated a good potential for cardiac tissue engineering applications.

Tissue engineering has also been explored in the development of skeletal muscle tissue. The selection of biomaterials for this application includes the focus on enhancing myoblast adhesion and promoting myogenic differentiation. Considering that skeletal myoblasts are naturally electroactive, the use of conductive materials, such as conducting polymers, in the skeletal muscle tissue engineering field has been extensively explored [74,75,76,77]. However, these polymers have shown to present not just toxicity but also reduced biodegradability. To overcome some of these challenges, Manchineella et al. [78] explored the use of silk fibroin combined with melanin in the development of an electroactive biocomposite scaffold. While silk fibroin (extracted from silkworm cocoons) has good biodegradability and biocompatibility, the conduction property of the biocomposite originated from the addition of melanin. Melanin, a natural polymeric pigment, displays conducting and antioxidant properties [79,80,81]. A blend of silk fibroin and melanin were electrospun in order to create a porous scaffold. Conductivity property of the biocomposite, in terms of resistance values, was evaluated using a resistivity probe at room temperature and results were compared with silk by itself. These results indicated that electrospun biocomposite scaffolds demonstrated 2.5 times decrease in resistance compared with silk, attributed to the porous nature of the scaffold and to the addition of melanin. Viability, cytotoxicity and proliferation of mouse myoblast cells seeded onto the biocomposite scaffolds were investigated. In addition to high cell viability, a homogeneous cell distribution was observed on the surface of the biocomposite. Myogenesis (formation of muscular tissue) was induced by creating serum starvation condition on the scaffolds containing confluent myoblasts. Multinucleated myotubes formation was observed due to myoblast fusion. Myotubes formed on silk scaffolds in absence of melanin were shorter, while presence of melanin on electrospun scaffolds resulted on well-defined, long and aligned myotubes. These results indicated that addition of melanin to silk fibroin improved myogenic differentiation of myoblast cells, demonstrating that this biocomposite has potential for applications in skeletal muscle tissue engineering.

Kim et al. [82] integrated 3D printing techniques on the development of a biocomposite scaffold consisting of PCL and collagen simulating microfibril muscle structure. In order to 3D print PCL microfibrils, poly(vinyl alcohol) (PVA) was mixed with PCL and a complex microscale-patterned 3D printed structure was created. PVA was then removed by immersing the 3D printed structure in distilled water for 24 h. Collagen was then deposited on the surface of the PCL fibers, in order to enhance myoblast adhesion. Myoblast proliferation and morphology were investigated by live/dead staining and MTT assay. Cell morphology was aligned parallel to the direction of the microfibrils, while structures without collagen did not induce alignment of cytoskeleton shape. In addition, higher cell proliferation was observed in the hybrid microfibrils struts. Gene expression factors illustrating myogenic differentiation were tracked using real-time polymerase chain reactions. Myoblasts cultured on the biocomposite structures demonstrated higher gene expression levels, indicating higher alignment and proliferation. Cells also infiltrated and proliferated between the microfibers of the scaffolds. These results illustrated that PCL/collagen-based scaffolds possess a good potential as substrates for muscle tissue regeneration.

Even though mimicking tissues in vitro has been one of the main goals of tissue engineering, vascularization remains a challenge. Lack of vascularization restricts the delivery of nutrients to cells by diffusion [83], which limits the final thickness and complexity of the scaffold. Focusing on the development of blood vessel scaffolds with applications to cardiovascular diseases, Badhe et al. [84] investigated the use of a biocomposite composed of chitosan and gelatin. The scaffolds showed a microporous bilayer structure, in which the inner layer provided a large surface area for cellular adhesion and proliferation, while the non-porous outer layer provided flexibility and elasticity. The porous inner layer was created by adding Eudragit® L100 particles to the hydrogel solution. This mixture was then transferred onto spinning plastic tubes and, after drying, the setup was immersed in a sodium hydroxide solution to dissolve the Eudragit® L100 particles, leaving behind a microporous tubular scaffold. A second layer of chitosan-gelatin hydrogel mixture was then applied to the tubular structure and allowed to dry, as illustrated in Figure 5d. The resulting scaffold demonstrated a final bilayer tubular structure. Human dermal fibroblast cells seeded in the tubular scaffolds spread and proliferated on the surface of the 3D structures, showing filopodia and reaching confluence by day 20. These results confirmed that the chitosan-gelatin tubular scaffolds can be considered a promising candidate for blood vessel tissue engineering.

Applications of biocomposites in tissue engineering also extents to the development of in vitro tumor models. Cancer is still a devastating disease that affects millions of people each year. In order to predict the response of cancer to treatment, studies are moving away from the 2D cell cultures into 3D models, which is an improvement considering that cells experience a 3D environment inside the body [85]. Therefore, 2D monolayers are unable to replicate this complexity [86] and, consequently, the translation of results obtained in 2D cell cultures are not very promising [87]. Zhao et al. [88] developed an in vitro cervical tumor model in order to investigate the response of paclitaxel in 3D constructs. The 3D constructs were fabricated by 3D printing Hela cells mixed with a biocomposite ink composed of gelatin, alginate and fibrinogen in a layer-by-layer fashion. Comparing with Hela 2D monolayers, Hela cells in the 3D constructs demonstrated the formation of round spheroids in addition to tight cell-cell connections within the hydrogel. Monolayers, on the other hand, showed flat and elongated morphology. Paclitaxel was added to the cell media of 3D scaffolds and 2D samples, in order to compare the response of the cells to this chemotherapy drug. While high cellular apoptosis was observed in both 2D and 3D samples, 3D printed Hela/hydrogel constructs demonstrated enhanced chemoresistance, illustrating the impact of dimensionality on the evaluation of effectiveness of chemotherapy.

In addition to providing a 3D environment, scaffolds should also match the mechanical properties of the tissue being modelled. Valente et al. [89] explored the use of a biocomposite mixture composed of collagen type I and gelatin methacryloyl in order to mimic healthy and cancerous breast tissues. By varying the ratio between these two polymers, different elastic moduli values were obtained for the biocomposites. Soft biocomposite scaffolds displayed an elastic modulus of ~200 Pa, while healthy breast tissue displayed elastic modulus ranging from 150 to 400 Pa. Stiff hydrogel mixture presented elastic modulus of ~5900 Pa, that was similar to values obtained for breast tumor samples (3–8 kPa) [90,91,92]. These biocomposite mixtures were then injected into microfluidic devices in order to create a breast-on-a-chip to evaluate the impact of matrix stiffness on the transport of GNP, as illustrated in Figure 5e. GNP are radiosensitizers that are capable of enhancing the effect of radiation therapy. Tracking the movement of fluorescently labeled GNP inside the microfluidic device demonstrated that there was a 77% decrease on the diffusion coefficient. The decrease in diffusive transport displayed the importance of biocomposite stiffness on transport of species. In addition to demonstrating high cell viability for MCF-7 breast cancer cells seeded on the scaffolds, diffusion results for GNP were similar to in vivo results obtained for Immunoglobulin G, highlighting the feasibility of application of this biocomposite in mimicking in vitro tumor and healthy breast ECM.

## 6. Conclusions

Biocomposites present great potential for application in a wide range of areas in biomedical engineering. Table 1 summarizes the highlighted applications of biocomposites discussed in this paper. In the case of synthetic bone grafts, biocomposites displayed good cell adhesion, proliferation and bone growth. In addition, high ALP levels were also observed, indicating the osteoinductive character of the biocomposites. Encouraging outcomes were also obtained for biocomposites containing PEEK, in which enhanced mechanical properties and high ALP activity level allows the application of these scaffolds to orthopedic/dental implants. Considering that wound dressings are required to be biocompatible, they contain a large quantity of water and provide gas exchange, biocomposite hydrogel materials are an appealing alternative to currently available wound dressings. Considering the diversity of the available biocompatible polymeric materials, there are many potential applications of biocomposites for tissue engineering. In summary, while promising results have been widely documented in the open literature, the integration of biocomposites in the human body requires further in vivo studies in order to assess the biocompatibility and non-toxicity of these scaffolds.

## Figures and Tables

**Table 1:** Summary of Biomedical Applications of Biocomposites.

Application	Biocomposite Composition	Reference
Bone Regeneration	Nanohydroxyapatite and glycol chitosan	[10]
	Hydroxyapatite and polymeric blend (fibroin, chitosan and agarose)	[11]
	Calcium silicate, zinc silicate and graphene oxide	[15]
	Collagen, silk fibroin and dECM	[26]
	Boron nitride and boron trioxide	[28]
	Nanohydroxyapatite, calcium sulfate and bioactive molecules	[32]
Orthopedic Implants	PEEK and graphene oxide	[39]
	CFRPEEK, nanohydroxyapatite, carboxymethyl, chitosan and bone forming peptide	[41]
	Polyphenylene sulfide and nanohydroxyapatite	[42]
	Polyimide and tantalum pentaoxide	[43]
	Hydroxyapatite, ceria nanoparticles and silver nanoparticles	[44]
Wound Healing	Polycaprolactone and gelatin	[54]
	Chitosan, polyethylene oxide and fibrinogen	[57]
	Collagen, alginate and silver nanoparticles	[60]
	Polyurethane, keratin and silver nanoparticles	[63]
	Collagen and dextran	[65]
Tissue Engineering	Fibrin, alginate and genipin	[68]
	PEDOT, chitosan and gelatin	[70]
	Polycaprolactone, silk fibroin and carbon nanotubes	[71]
	Silk fibroin and melanin	[78]
	Polycaprolactone and collagen	[82]
	Gelatin, alginate and fibrinogen	[88]
	Collagen type I and gelatin methacryloyl	[89]

## References

[1] Dorozhkin S.V. (2011). Biocomposites and hybrid biomaterials based on calcium orthophosphates. Biomatter.

[2] Haraguchi K. (2015). Biocomposites. Encyclopedia of Polymeric Nanomaterials.

[3] Yunus Basha R., Sampath S.K., Doble M. (2015). Design of biocomposite materials for bone tissue regeneration. Mater. Sci. Eng. C.

[4] Bružauskaitė I., Bironaitė D., Bagdonas E., Bernotienė E. (2016). Scaffolds and cells for tissue regeneration: Different scaffold pore sizes—Different cell effects. Cytotechnology.

[5] Tajbakhsh S., Hajiali F. (2017). A comprehensive study on the fabrication and properties of biocomposites of poly (lactic acid)/ceramics for bone tissue engineering. Mater. Sci. Eng. C.

[6] Swetha M., Sahithi K., Moorthi A., Srinivasan N., Ramasamy K., Selvamurugan N. (2010). Biocomposites containing natural polymers and hydroxyapatite for bone tissue engineering. Int. J. Biol. Macromol..

[7] Regí M.V., Navarrete D.A. (2015). Biological Apatites in Bone and Teeth. Nanoceramics in Clinical Use: From Materials to Applications.

[8] Sanchez C., Arribart H., Guille M.M.G. (2005). Biomimetism and bioinspiration as tools for the design of innovative materials and systems. Nat. Mater..

[9] Chiara G., Letizia F., Lorenzo F., Edoardo S., Diego S., Stefano S., Eriberto B., Barbara Z. (2012). Nanostructured biomaterials for tissue engineered bone tissue reconstruction. Int. J. Mol. Sci..

[10] Dumont V.C., Mansur H.S., Mansur A.A.P., Carvalho S.M., Capanema N.S.V., Barrioni B.R. (2016). Glycol chitosan/nanohydroxyapatite biocomposites for potential bone tissue engineering and regenerative medicine. Int. J. Biol. Macromol..

[11] Raina D.B., Isaksson H., Teotia A.K., Lidgren L., Tägil M., Kumar A. (2016). Biocomposite macroporous cryogels as potential carrier scaffolds for bone active agents augmenting bone regeneration. J. Control. Release.

[12] Belfrage O., Flivik G., Sundberg M., Kesteris U., Tägil M. (2011). Local treatment of cancellous bone grafts with BMP-7 and zoledronate increases both the bone formation rate and bone density: A bone chamber study in rats. Acta Orthop..

[13] Mathavan N., Bosemark P., Isaksson H., Tägil M. (2013). Investigating the synergistic efficacy of BMP-7 and zoledronate on bone allografts using an open rat osteotomy model. Bone.

[14] Bosemark P., Perdikouri C., Pelkonen M., Isaksson H., Tägil M. (2015). The masquelet induced membrane technique with BMP and a synthetic scaffold can heal a rat femoral critical size defect. J. Orthop. Res..

[15] Xiong K., Wu T., Fan Q., Chen L., Yan M. (2017). Novel Reduced Graphene Oxide/Zinc Silicate/Calcium Silicate Electroconductive Biocomposite for Stimulating Osteoporotic Bone Regeneration. ACS Appl. Mater. Interfaces.

[16] Lin K., Xia L., Li H., Jiang X., Pan H., Xu Y., Lu W.W., Zhang Z., Chang J. (2013). Enhanced osteoporotic bone regeneration by strontium-substituted calcium silicate bioactive ceramics. Biomaterials.

[17] Weng L., Boda S.K., Teusink M.J., Shuler F.D., Li X., Xie J. (2017). Binary Doping of Strontium and Copper Enhancing Osteogenesis and Angiogenesis of Bioactive Glass Nanofibers while Suppressing Osteoclast Activity. ACS Appl. Mater. Interfaces.

[18] Xia L., Yin Z., Mao L., Wang X., Liu J., Jiang X., Zhang Z., Lin K., Chang J., Fang B. (2016). Akermanite bioceramics promote osteogenesis, angiogenesis and suppress osteoclastogenesis for osteoporotic bone regeneration. Sci. Rep..

[19] Zhang W., Zhao F., Huang D., Fu X., Li X., Chen X. (2016). Strontium-Substituted Submicrometer Bioactive Glasses Modulate Macrophage Responses for Improved Bone Regeneration. ACS Appl. Mater. Interfaces.

[20] Mao L., Xia L., Chang J., Liu J., Jiang L., Wu C., Fang B. (2017). The synergistic effects of Sr and Si bioactive ions on osteogenesis, osteoclastogenesis and angiogenesis for osteoporotic bone regeneration. Acta Biomater..

[21] Yamaguchi M. (2010). Role of nutritional zinc in the prevention of osteoporosis. Mol. Cell. Biochem..

[22] Carlisle E.M. (1970). Silicon: A possible factor in bone calcification. Science.

[23] Zhang N., Molenda J.A., Fournelle J.H., Murphy W.L., Sahai N. (2010). Effects of pseudowollastonite (CaSiO3) bioceramic on in vitro activity of human mesenchymal stem cells. Biomaterials.

[24] Xu S., Lin K., Wang Z., Chang J., Wang L., Lu J., Ning C. (2008). Reconstruction of calvarial defect of rabbits using porous calcium silicate bioactive ceramics. Biomaterials.

[25] Rodriguez y Baena R., Rizzo S., Graziano A., Lupi S.M. (2016). Bone Regeneration in Implant Dentistry: Role of Mesenchymal Stem Cells. A Textbook of Advanced Oral and Maxillofacial Surgery Volume 3.

[26] Lee H., Yang G.H., Kim M., Lee J.Y., Huh J.T., Kim G.H. (2017). Fabrication of micro/nanoporous collagen/dECM/silk-fibroin biocomposite scaffolds using a low temperature 3D printing process for bone tissue regeneration. Mater. Sci. Eng. C.

[27] Karageorgiou V., Kaplan D. (2005). Porosity of 3D biomaterial scaffolds and osteogenesis. Biomaterials.

[28] Gautam C., Chakravarty D., Gautam A., Tiwary C.S., Woellner C.F., Mishra V.K., Ahmad N., Ozden S., Jose S., Biradar S. (2018). Synthesis and 3D Interconnected Nanostructured h-BN-Based Biocomposites by Low-Temperature Plasma Sintering: Bone Regeneration Applications. ACS Omega.

[29] Huang J.L., Nayak P.K. (2014). Strengthening alumina ceramic matrix nanocomposites using spark plasma sintering. Advances in Ceramic Matrix Composites.

[30] Santanach J.G., Weibel A., Estourns C., Yang Q., Laurent C., Peigney A. (2011). Spark plasma sintering of alumina: Study of parameters, formal sintering analysis and hypotheses on the mechanism(s) involved in densification and grain growth. Acta Mater..

[31] Shah A.M., Jung H., Skirboll S. (2014). Materials used in cranioplasty: A history and analysis. Neurosurg. Focus.

[32] Teotia A.K., Raina D.B., Singh C., Sinha N., Isaksson H., Tägil M., Lidgren L., Kumar A. (2017). Nano-Hydroxyapatite Bone Substitute Functionalized with Bone Active Molecules for Enhanced Cranial Bone Regeneration. ACS Appl. Mater. Interfaces.

[33] Peltier L.F. (1959). The use of plaster of paris to fill large defects in bone. A preliminary report. Am. J. Surg..

[34] Cobb A.G., Schmalzreid T.P. (2006). The clinical significance of metal ion release from cobalt-chromium metal-on-metal hip joint arthroplasty. Proc. Inst. Mech. Eng. Part H J. Eng. Med..

[35] Campbell J.R., Estey M.P. (2013). Metal release from hip prostheses: Cobalt and chromium toxicity and the role of the clinical laboratory. Clin. Chem. Lab. Med..

[36] Ryan G., Pandit A., Apatsidis D.P. (2006). Fabrication methods of porous metals for use in orthopaedic applications. Biomaterials.

[37] Abu Bakar M.S., Cheng M.H.W., Tang S.M., Yu S.C., Liao K., Tan C.T., Khor K.A., Cheang P. (2003). Tensile properties, tension-tension fatigue and biological response of polyetheretherketone-hydroxyapatite composites for load-bearing orthopedic implants. Biomaterials.

[38] Ma R., Tang S., Tan H., Qian J., Lin W., Wang Y., Liu C., Wei J., Tang T. (2014). Preparation, characterization, in vitro bioactivity and cellular responses to a polyetheretherketone bioactive composite containing nanocalcium silicate for bone repair. ACS Appl. Mater. Interfaces.

[39] Ouyang L., Deng Y., Yang L., Shi X., Dong T., Tai Y., Yang W., Chen Z.G. (2018). Graphene-Oxide-Decorated Microporous Polyetheretherketone with Superior Antibacterial Capability and In Vitro Osteogenesis for Orthopedic Implant. Macromol. Biosci..

[40] Luo Y., Shen H., Fang Y., Cao Y., Huang J., Zhang M., Dai J., Shi X., Zhang Z. (2015). Enhanced proliferation and osteogenic differentiation of mesenchymal stem cells on graphene oxide-incorporated electrospun poly(lactic-co-glycolic acid) nanofibrous mats. ACS Appl. Mater. Interfaces.

[41] Xu A., Zhou L., Deng Y., Chen X., Xiong X., Deng F., Wei S. (2016). A carboxymethyl chitosan and peptide-decorated polyetheretherketone ternary biocomposite with enhanced antibacterial activity and osseointegration as orthopedic/dental implants. J. Mater. Chem. B.

[42] Deng Y., Yang Y., Ma Y., Fan K., Yang W., Yin G. (2017). Nano-hydroxyapatite reinforced polyphenylene sulfide biocomposite with superior cytocompatibility and in vivo osteogenesis as a novel orthopedic implant. RSC Adv..

[43] Asadullah S., Wu H., Mei S., Wang D., Pan Y., Wang D., Zhao J., Wei J. (2019). Preparation, characterization, in vitro bioactivity and rBMSCs responses to tantalum pentoxide/polyimide biocomposites for dental and orthopedic implants. Compos. Part B Eng..

[44] Pandey A., Midha S., Sharma R.K., Maurya R., Nigam V.K., Ghosh S., Balani K. (2018). Antioxidant and antibacterial hydroxyapatite-based biocomposite for orthopedic applications. Mater. Sci. Eng. C.

[45] Afzal M.A.F., Kalmodia S., Kesarwani P., Basu B., Balani K. (2013). Bactericidal effect of silver-reinforced carbon nanotube and hydroxyapatite composites. J. Biomater. Appl..

[46] Mariam J., Dongre P.M., Kothari D.C. (2011). Study of interaction of silver nanoparticles with bovine serum albumin using fluorescence spectroscopy. J. Fluoresc..

[47] Tanaka M., Motomura T., Kawada M., Anzai T., Yuu K., Shiroya T., Shimura K., Onishi M., Mochizuki A. (2000). Blood compatible aspects of poly(2-methoxyethylacrylate) (PMEA)-relationship between protein adsorption and platelet adhesion on PMEA surface. Biomaterials.

[48] Jayakumar R., Prabaharan M., Sudheesh Kumar P.T., Nair S.V., Tamura H. (2011). Biomaterials based on chitin and chitosan in wound dressing applications. Biotechnol. Adv..

[49] Aramwit P. (2016). Introduction to biomaterials for wound healing. Wound Healing Biomaterials.

[50] Saghazadeh S., Rinoldi C., Schot M., Kashaf S.S., Sharifi F., Jalilian E., Nuutila K., Giatsidis G., Mostafalu P., Derakhshandeh H. (2018). Drug delivery systems and materials for wound healing applications. Adv. Drug Deliv. Rev..

[51] Sen C.K. (2009). Wound healing essentials: Let there be oxygen. Wound Repair Regen..

[52] Lin S., Sangaj N., Razafiarison T., Zhang C., Varghese S. (2011). Influence of physical properties of biomaterials on cellular behavior. Pharm. Res..

[53] Suarato G., Bertorelli R., Athanassiou A. (2018). Borrowing from nature: Biopolymers and biocomposites as smart wound care materials. Front. Bioeng. Biotechnol..

[54] Anjum F., Agabalyan N.A., Sparks H.D., Rosin N.L., Kallos M.S., Biernaskie J. (2017). Biocomposite nanofiber matrices to support ECM remodeling by human dermal progenitors and enhanced wound closure. Sci. Rep..

[55] Nair L.S., Laurencin C.T. (2006). Polymers as biomaterials for tissue engineering and controlled drug delivery. Adv. Biochem. Eng. Biotechnol..

[56] Jabbari E. (2011). Bioconjugation of hydrogels for tissue engineering. Curr. Opin. Biotechnol..

[57] Yuan T.T., DiGeorge Foushee A.M., Johnson M.C., Jockheck-Clark A.R., Stahl J.M. (2018). Development of Electrospun Chitosan-Polyethylene Oxide/Fibrinogen Biocomposite for Potential Wound Healing Applications. Nanoscale Res. Lett..

[58] Pierce G.F., Mustoe T.A., Altrock B.W., Deuel T.F., Thomason A. (1991). Role of platelet-derived growth factor in wound healing. J. Cell. Biochem..

[59] Raafat D., Sahl H.G. (2009). Chitosan and its antimicrobial potential—A critical literature survey. Microb. Biotechnol..

[60] Zhang H., Peng M., Cheng T., Zhao P., Qiu L., Zhou J., Lu G., Chen J. (2018). Silver nanoparticles-doped collagen–alginate antimicrobial biocomposite as potential wound dressing. J. Mater. Sci..

[61] Szekalska M., Puciłowska A., Szymańska E., Ciosek P., Winnicka K. (2016). Alginate: Current Use and Future Perspectives in Pharmaceutical and Biomedical Applications. Int. J. Polym. Sci..

[62] Dowling M.B., Chaturvedi A., MacIntire I.C., Javvaji V., Gustin J., Raghavan S.R., Scalea T.M., Narayan M. (2016). Determination of efficacy of a novel alginate dressing in a lethal arterial injury model in swine. Injury.

[63] Wang Y., Li P., Xiang P., Lu J., Yuan J., Shen J. (2016). Electrospun polyurethane/keratin/AgNP biocomposite mats for biocompatible and antibacterial wound dressings. J. Mater. Chem. B.

[64] Lakshman L.R., Shalumon K.T., Nair S.V., Jayakumar R., Nair S.V. (2010). Preparation of silver nanoparticles incorporated electrospun polyurethane nano-fibrous mat for wound dressing. J. Macromol. Sci. Part A Pure Appl. Chem..

[65] Roussakis E., Ortines R.V., Pinsker B.L., Mooers C.T., Evans C.L., Miller L.S., Calderón-Colón X. (2019). Theranostic biocomposite scaffold membrane. Biomaterials.

[66] Eltom A., Zhong G., Muhammad A. (2019). Scaffold Techniques and Designs in Tissue Engineering Functions and Purposes: A Review. Adv. Mater. Sci. Eng..

[67] Celikkin N., Rinoldi C., Costantini M., Trombetta M., Rainer A., Święszkowski W. (2017). Naturally derived proteins and glycosaminoglycan scaffolds for tissue engineering applications. Mater. Sci. Eng. C.

[68] Lee C., Abelseth E., de la Vega L., Willerth S.M. (2019). Bioprinting a novel glioblastoma tumor model using a fibrin-based bioink for drug screening. Mater. Today Chem..

[69] Lee C., Robinson M., Willerth S.M. (2018). Direct Reprogramming of Glioblastoma Cells into Neurons Using Small Molecules. ACS Chem. Neurosci..

[70] Wang S., Sun C., Guan S., Li W., Xu J., Ge D., Zhuang M., Liu T., Ma X. (2017). Chitosan/gelatin porous scaffolds assembled with conductive poly (3,4-ethylenedioxythiophene) nanoparticles for neural tissue engineering. J. Mater. Chem. B.

[71] Wu Y., Wang L., Guo B., Ma P.X. (2017). Interwoven Aligned Conductive Nanofiber Yarn/Hydrogel Composite Scaffolds for Engineered 3D Cardiac Anisotropy. ACS Nano.

[72] Hanley P.J., Young A.A., LeGrice I.J., Edgar S.G., Loiselle D.S. (1999). 3-Dimensional configuration of perimysial collagen fibres in rat cardiac muscle at resting and extended sarcomere lengths. J. Physiol..

[73] Macchiarelli G., Ohtani O., Nottola S.A., Stallone T., Camboni A., Prado I.M., Motta P.M. (2002). A micro-anatomical model of the distribution of myocardial endomysial collagen. Histol. Histopathol..

[74] Sirivisoot S., Harrison B.S. (2011). Skeletal myotube formation enhanced by electrospun polyurethane carbon nanotube scaffolds. Int. J. Nanomedicine.

[75] Thrivikraman G., Mallik P.K., Basu B. (2013). Substrate conductivity dependent modulation of cell proliferation and differentiation invitro. Biomaterials.

[76] Broda C.R., Lee J.Y., Sirivisoot S., Schmidt C.E., Harrison B.S. (2011). A chemically polymerized electrically conducting composite of polypyrrole nanoparticles and polyurethane for tissue engineering. J. Biomed. Mater. Res. Part A.

[77] Chen M.C., Sun Y.C., Chen Y.H. (2013). Electrically conductive nanofibers with highly oriented structures and their potential application in skeletal muscle tissue engineering. Acta Biomater..

[78] Manchineella S., Thrivikraman G., Khanum K.K., Ramamurthy P.C., Basu B., Govindaraju T. (2016). Pigmented Silk Nanofibrous Composite for Skeletal Muscle Tissue Engineering. Adv. Healthc. Mater..

[79] Ju K.Y., Lee Y., Lee S., Park S.B., Lee J.K. (2011). Bioinspired polymerization of dopamine to generate melanin-like nanoparticles having an excellent free-radical-scavenging property. Biomacromolecules.

[80] Bothma J.P., de Boor J., Divakar U., Schwenn P.E., Meredith P. (2008). Device-Quality Electrically Conducting Melanin Thin Films. Adv. Mater..

[81] Kim E., Liu Y., Leverage W.T., Yin J.J., White I.M., Bentley W.E., Payne G.F. (2014). Context-dependent redox properties of natural phenolic materials. Biomacromolecules.

[82] Kim W., Kim M., Kim G.H. (2018). 3D-Printed Biomimetic Scaffold Simulating Microfibril Muscle Structure. Adv. Funct. Mater..

[83] Valente K.P., Khetani S., Kolahchi A.R., Sanati-Nezhad A., Suleman A., Akbari M. (2017). Microfluidic technologies for anticancer drug studies. Drug Discov. Today.

[84] Badhe R.V., Bijukumar D., Chejara D.R., Mabrouk M., Choonara Y.E., Kumar P., du Toit L.C., Kondiah P.P.D., Pillay V. (2017). A composite chitosan-gelatin bi-layered, biomimetic macroporous scaffold for blood vessel tissue engineering. Carbohydr. Polym..

[85] Papera Valente K., Singh Thind S., Suleman A., Brolo A. Merging micro and nano: Study of transport of gold nanoparticles inside a tumor microenvironment-on-a-chip. Proceedings of the SPIE—The International Society for Optical Engineering.

[86] Padrón J.M., Van Der Wilt C.L., Smid K., Smitskamp-Wilms E., Backus H.H.J., Pizao P.E., Giaccone G., Peters G.J. (2000). The multilayered postconfluent cell culture as a model for drug screening. Crit. Rev. Oncol. Hematol..

[87] Pampaloni F., Reynaud E.G., Stelzer E.H.K. (2007). The third dimension bridges the gap between cell culture and live tissue. Nat. Rev. Mol. Cell Biol..

[88] Zhao Y., Yao R., Ouyang L., Ding H., Zhang T., Zhang K., Cheng S., Sun W. (2014). Three-dimensional printing of Hela cells for cervical tumor model in vitro. Biofabrication.

[89] Valente K.P., Thind S.S., Akbari M., Suleman A., Brolo A.G. (2019). Collagen Type I-Gelatin Methacryloyl Composites: Mimicking the Tumor Microenvironment. ACS Biomater. Sci. Eng..

[90] Acerbi I., Cassereau L., Dean I., Shi Q., Au A., Park C., Chen Y.Y., Liphardt J., Hwang E.S., Weaver V.M. (2015). Human breast cancer invasion and aggression correlates with ECM stiffening and immune cell infiltration. Integr. Biol..

[91] Asghar W., El Assal R., Shafiee H., Pitteri S., Paulmurugan R., Demirci U. (2015). Engineering cancer microenvironments for in vitro 3-D tumor models. Mater. Today.

[92] Paszek M.J., Zahir N., Johnson K.R., Lakins J.N., Rozenberg G.I., Gefen A., Reinhart-King C.A., Margulies S.S., Dembo M., Boettiger D. (2005). Tensional homeostasis and the malignant phenotype. Cancer Cell.

